# Professional fulfillment and parenting work-life balance in female physicians in Basic Sciences and medical research: a nationwide cross-sectional survey of all 80 medical schools in Japan

**DOI:** 10.1186/s12960-017-0241-0

**Published:** 2017-09-15

**Authors:** Yuka Yamazaki, Takanori Uka, Eiji Marui

**Affiliations:** 1 Department of Medical Education, Tokyo Medical University, 6-7-1, Nishi-Shinjuku, Shinjuku-ku, Tokyo Japan; 2 Department of Neurophysiology, Juntendo University Graduate School of Medicine, Tokyo, Japan; 3 Department of Human Arts Sciences, University of Human Arts and Sciences, Saitama, Japan

**Keywords:** Basic Sciences, Research, Parental duties, Japan, Female physicians, Work-life balance

## Abstract

**Background:**

In Japan, the field of Basic Sciences encompasses clinical, academic, and translational research, as well as the teaching of medical sciences, with both an MD and PhD typically required. In this study, it was hypothesized that the characteristics of a Basic Sciences career path could offer the professional advancement and personal fulfillment that many female medical doctors would find advantageous. Moreover, encouraging interest in Basic Sciences could help stem shortages that Japan is experiencing in medical fields, as noted in the three principal contributing factors: premature resignation of female clinicians, an imbalance of female physicians engaged in research, and a shortage of medical doctors in the Basic Sciences. This study examines the professional and personal fulfillment expressed by Japanese female medical doctors who hold positions in Basic Sciences. Topics include career advancement, interest in medical research, and greater flexibility for parenting.

**Methods:**

A cross-sectional questionnaire survey was distributed at all 80 medical schools in Japan, directed to 228 female medical doctors whose academic rank was assistant professor or higher in departments of Basic Sciences in 2012. Chi-square tests and the binary logistic regression model were used to investigate the impact of parenthood on career satisfaction, academic rank, salary, etc.

**Results:**

The survey response rate of female physicians in Basic Sciences was 54.0%. Regardless of parental status, one in three respondents cited research interest as their rationale for entering Basic Sciences, well over twice other motivations. A majority had clinical experience, with clinical duties maintained part-time by about half of respondents and particularly parents. Only one third expressed afterthoughts about relinquishing full-time clinical practice, with physicians who were parents expressing stronger regrets. Parental status had little effect on academic rank and income within the Basic Sciences,

**Conclusion:**

Scientific curiosity and a desire to improve community health are hallmarks of those choosing a challenging career in medicine. Therefore, it is unsurprising that interest in research is the primary motivation for a female medical doctor to choose a career in Basic Sciences. Additionally, as with many young professionals with families, female doctors seek balance in professional and private lives. Although many expressed afterthoughts relinquishing a full-time clinical practice, mothers generally benefited from greater job flexibility, with little significant effect on career development and income as Basic Scientists.

**Electronic supplementary material:**

The online version of this article (10.1186/s12960-017-0241-0) contains supplementary material, which is available to authorized users.

## Background

In Japan, Basic Sciences encompass clinical, academic, and translational research, as well as teaching medical sciences, with an MD and PhD [1] typically required. The hypothesis of this study is that a Basic Sciences career path could offer professional advancement and personal fulfillment that many female physicians would find advantageous. Additionally, encouraging interest in Basic Sciences could help stem shortages that Japan is experiencing in medical fields, as noted in three principal contributing factors.

First, a trend of early-career resignations by female physicians in their late 20s and 30s [2] in Japan has resulted in significant decline of workforce participation. In the last 30 years, the proportion of female physicians increased from 10.4% in 1984 [3] to 20.4% in 2014 [4]. Yet, government reports reveal that labor force participation rates of female physicians fall to 75% within 9 years after graduation [5], with childbirth and parental duties cited as principal reasons [2, 6]. In Japan, domestic responsibilities, and especially parental duties, fall on women’s shoulders much more heavily than men’s, even when the wife is the physician [7]. Therefore, with motherhood, female physicians find managing work and parental duties difficult. Early-career departures lead to shortages in clinical fields, oppressive working conditions, and excessive hours for remaining physicians.

The first author’s (YY) qualitative study concludes that young physicians desire satisfaction in career and personal lives [8]. Thus, recruitment strategies for hiring and retaining young physicians must reflect expected work-life-balance assessments by providing accurate and detailed information about each specialty, including anticipated lifestyle demands [8].

Second, among Japan’s medical schools, an imbalance of female physicians engaged in research is evident, with 2.6% in 2012 [8] compared to 20% in the USA [9]. At one national medical school in 2013, 40 females and 326 males ranked as assistant professor or higher [10]. The proportion of female researchers in Japan is the lowest among developed countries at 14% [11]. While these numbers suggest obstacles in developing research careers, childbirth and parental duties also hinder academic career development for female physicians [12]. Without appropriate measures, females face fewer opportunities to advance academic careers in an appropriate time frame.

Third, there are shortages of physicians in Basic Sciences, which include such pre-clinical fields as Anatomy, Physiology, Biochemistry, Pathology, and Immunology. Basic research has traditionally been led by researchers with both an MD and PhD [1], so these shortages could threaten a personnel crisis in Japan [1]. Great intelligence, resilience, and dedication are required for academic and clinical preparation as a physician [13], which also holds true for a medical researcher or professor teaching in medical school [14]. The difficulty of working a clinician’s extended hours and traditionally raising children as a Japanese wife may influence medical doctor shortages [15]. In fact, one survey comparing work-life balance in Japan with the USA and Hong Kong revealed that the ratio of full-time female surgeons in Japan was reduced to less than 50% after childbirth, whereas this ratio in the USA and Hong Kong was about 80 and 86%, respectively [16]. Japanese female doctors with children may experience considerable guilt when career demands compete with parental duties, feeling less connected to the “ideal” Japanese vision of mothers and wives [17], even as they try to adhere to domestic gender roles. For example, the abovementioned study reported that approximately half of female surgeons in the USA and Hong Kong consider both genders as equals at home, but that number falls to 25% in Japan, where 50% also believe that women take more responsibilities than men at home [16].

One historical report details Japan’s traditional culture of shame, which causes Japanese to be excessively sensitive to others’ evaluations, while generally considering their own opinions inconsequential [18]. Given that Japanese society values the role of housewife and mother highly, working mothers may be overly concerned about evaluations by colleagues or managers and feel shame when they cannot meet the expectations of others [18, 19]. This shame is endured when female physicians are scheduled fewer hours than male doctors [15], unable to accept themselves when they inconvenience colleagues [15]. In making an effort to work as hard as male colleagues and female colleagues without children [17], they eventually feel inadequate or become “burned out” in the workplace. They may often experience “superwoman” syndrome and are torn between workplace and family [20].

Thus, critical shortages of Basic Sciences professionals can be addressed with the belief that, with or without part-time clinical work, a career in Basic Sciences can provide a rewarding alternative for women who value time for family, more flexibility in hours, and control over workflow. Additionally, the entry of more female physicians into Basic Sciences departments would not only reduce shortages in the field, but also reduce disparities for female researchers.

However, only limited research has examined the demographic of female physicians in Basic Sciences departments, including those abroad. Therefore, because female medical students and physicians lack sufficient opportunities to observe the work and lifestyle of physicians, and particularly parents, in Basic Sciences, the career is rarely considered.

In 2011, the authors conducted a questionnaire survey focused on physicians at one medical school in Tokyo to investigate characteristics and development of physicians in Basic Sciences [21]. With an overall response rate of 87%, 27% of respondents were female. Among physicians, women entered Basic Sciences at a younger age than men, with significantly more women advocating for medical students to conduct research. Moreover, flexible working hours and no night duties were mentioned as benefits of working in Basic Sciences [21], and promoting these advantages could be an effective recruitment strategy [21].

For the current study, a questionnaire survey was mailed to all Japanese medical schools in October, 2012, targeting all female physicians in Basic Sciences departments with a rank of assistant professor or higher. The hypothesis is that female physicians with an interest and aptitude for research or teaching could find greater career satisfaction in Basic Sciences and a healthy work-life balance without suffering a decline in career and salary advancement after childbirth. Our objective for this study is to test the hypothesis by investigating the impact of motherhood on career satisfaction, academic rank, salary, etc. for female physicians who have chosen a Basic Sciences career, aided by gathering relevant demographic data.

## Methods

### Study subjects

This cross-sectional questionnaire survey was distributed at all 80 medical schools in Japan, focusing on 228 female physicians whose academic rank was assistant professor or higher in Basic Sciences departments. Their gender, degree, and academic rank were evaluated by information provided by each university’s website. All female physicians satisfying the above conditions were included. The definition of Basic Sciences is based on the science courses that the Japanese Ministry of Education, Culture, Sports, Science and Technology (MEXT) requires medical students to study, including anatomy, physiology, biochemistry, pathology, immunology, bacteriology, pharmacology, hygieiology, public health, and forensic medicine [22]. Basic Sciences departments were identified according to each medical school’s policy.

### Data collection

The anonymous questionnaires were distributed to subjects’ offices, as posted on the medical schools’ website, and collected by mail. Upon receipt, completed questionnaires were immediately labeled with an identification number and then processed in a delinked anonymous manner. Study data was collected in October 2012, over a period of 1 month. Reminder mails, which increased response rate, were sent to subjects 1 week before the deadline.

### Questionnaire contents

The 15-item questionnaire (Additional file 1) asked for the following: current age, number of years after medical school that participant entered Basic Sciences, motivation for entering Basic Sciences, medical specialty, academic rank, if full-time clinician in early career, if presently working as a part-time clinician, if afterthoughts were experienced in relinquishing full-time clinical practice, childbirth experience status, opinion on desirability of a Basic Sciences career for female physicians (and reasoning), if modifications in schedule or work environment received as “consideration” for being female (either positive, like more flexible hours, or negative, like losing a desired position), if a personal effort was made to inspire medical students about research, career choice satisfaction, and annual income.

### Analysis method

The independent variable was whether respondents had a child, and dependent variables were other questionnaire items except age and specialty. Chi-square tests were conducted to examine associations between having a child and other variables. However, age was excluded from analysis, since older women were more likely to already have children, compared with younger ones. In addition, specialty was excluded from analysis. Specialties were so diverse that sample size in each category would be too small to be statistically significant and, in some cases, could identify a participant. Moreover, an independent *t* test was conducted to examine the differential in number of years after medical school graduation that respondents with or without children entered Basic Sciences. To adjust for clinical experience, the binary logistic regression model was used with dependent variables to account for childbirth experience and afterthoughts relinquishing full-time clinical practice. The binary logistic regression model was based on this model: log *p*/1 − *p* = *β*1*X*1 + *β*2*X*2 +  ・  ・  ・  + *βpXp* + *β* 0, with the dependent variable as *afterthoughts relinquishing full-time clinical practice* (*Yes, often* and *Yes, sometimes* considered as *Yes* and *Almost never* and *Not at all* considered as *No*). The confounding factor was clinical experience. The statistical software package SPSS version 20 was used for all analyses. *P* values less than 0.05 were considered statistically significant. Results of logistic regression models were presented as odds ratios with 95% confidence intervals (CI), with incomplete data excluded from all analyses.

## Results

The questionnaire was returned by 123 of the 228 female physicians (response rate 54.0%). However, 2 of 123 were not working full-time in Basic Sciences, and therefore excluded from the analysis, with 121 analyzed. Demographic data for the respondents are shown in Table 1, where 43.8% (53/121) were in their 40s, 86.8% (105/121) entered Basic Sciences within 10 years of graduation, the most common reason for entering Basic Sciences was interest in research (32.3%), and the second most common reason was better work-life balance (14.2%). Pathologists accounted for 34.7% (42/121) of respondents, and those in professor rank accounted for 14.9% (18/121). In terms of clinical experience, 83.5% (101/121) had been a full-time physician, 47.1% (57/121) were currently engaged in clinical work as part-time physicians, and 31.4% (38/121) expressed afterthoughts relinquishing full-time clinical practice (often and sometimes yes). With regard to motherhood, 49.6% (60/121) had children; 45.5% (55/121) felt that the Basic Sciences were an advantageous career choice for female physicians (yes and generally yes), with the most common reason being the better work-life balance (42.2%); and 43.8% (102/121) were given considerations deemed favorable for women, such as allowance for early return or a flexible work schedule for parental duties. Fully 92.5% (112/121) were satisfied with their career choice (yes and generally yes), and the most commonly reported incomes in Yen (October 2012 exchange rate 1 GBP = 127 JPY) were 8.0–10.0 million JPY (63.0K–78.7K GBP) and 10.0–12.0 million JPY (78.7K–94.5K GBP).

**Table 1:** Respondent attributes

	Number	Percent
Age		
20s	4	3.3
30s	33	27.3
40s	53	43.8
50s	23	19.0
60s	7	5.8
No answer	1	0.8
Years after medical school graduation you entered Basic Sciences field?		
Under 1 year	33	27.3
2–5 years	43	35.5
6–10 years	29	24.0
Over 11 years	15	12.3
No answer	1	0.8
Motivation to enter Basic Sciences (multiple answers)		
Interest in research	75	32.3
Interest in disorders	28	12.1
Continued in Basic Sciences after receiving PhD	23	9.9
Career advancement	13	5.6
Wanted to leave clinical work	10	4.3
No particular reason	15	6.4
Better work-life balance	33	14.2
Others	33	14.2
No answer	2	0.9
(*n* = 121)		
Specialty		
Pathology	42	34.7
Physiology	17	14.0
Microbiology	4	3.3
Anatomy	8	6.6
Social medicine	22	18.2
Forensic medicine	6	5.0
Biochemistry	2	1.7
Immunology	4	3.3
Pharmacology	6	5.0
Genetics	3	2.5
Others	5	4.1
No answer	2	1.7
Academic rank		
Professor	18	14.9
Associate Professor	29	24.0
Senior Assistant Professor	26	21.5
Junior Assistant Professor	44	36.4
Others	4	3.3
Clinical experience		
Yes	101	83.5
No	20	16.5
Presently conducting clinical work regularly?		
Yes	57	47.1
No	63	52.1
No answer	1	0.8
Afterthoughts relinquishing full-time clinician practice?		
Yes, often	8	6.6
Yes, sometimes	30	24.8
Almost never	52	43.0
Not at all	24	19.8
No answer	7	5.8
Birth experience		
Yes	60	49.6
No	42	34.7
No answer	19	15.7
Is Basic Sciences a good fit with female MDs’ career goals?		
Yes	22	18.2
Generally yes	33	27.3
Not sure	46	38.0
Generally no	13	10.7
No	1	0.8
No answer	6	5.0
Reasons Basic Sciences are advantageous (multiple answer) (*n* = 56)		
Having time to take care of children	22	19.0
Not physically demanding	17	14.7
Managing work and private life	49	42.2
Basic Sciences compatible with female identity	22	19.0
Others	4	3.4
No answer	2	1.7
Received modified working considerations as a woman?		
Favorable considerations	53	43.8
Unfavorable considerations	8	6.6
No	44	36.4
Not sure	13	10.7
No answer	3	2.5
Made efforts to inspire student interest in research		
Yes	51	42.1
Generally yes	51	42.1
Generally no	13	10.7
No	3	2.5
Not engaged in lectures or practical trainings	2	1.7
No answer	1	0.8
Satisfaction with your career choice		
Yes	81	66.9
Generally yes	31	25.6
Not sure	7	5.8
Generally no	1	0.8
No answer	1	0.8
Total salary of previous year in million JPY (thousand GBP, October 2012) 1 GBP = 127 JPY		
< 4.0 (< 31.5)	1	0.8
4.0–6.0 (31.5–47.2)	11	9.1
6.0–8.0 (47.2–63.0)	23	19.0
8.0–10.0 (63.0–78.7)	35	28.9
10.0–12.0 (78.7–94.5)	35	28.9
12.0–14.0 (94.5–110.2)	8	6.6
14.0–16.0 (110.2–126.0)	3	2.5
16.0–18.0 (126.0–141.7)	1	0.8
No answer	4	3.3

Results of *χ* ^2^ tests, whether indicating significant differences or other meaningful results without significant differences, are shown in Table 2. Those with children were more likely to report better work-life balance as their motivation to enter Basic Sciences departments and also more likely to receive favorable considerations as women (*P* < 0.05). In addition, results of *χ* ^2^ tests showed that women with children were more likely both to engage in clinical work part-time and experience afterthoughts relinquishing full-time clinical practice (*P* < 0.05). There were no significant differences between childbirth experience and academic rank or salary (*P* = 0.6 and *P* = 0.95, respectively).

**Table 2:** The influence of motherhood on female physicians' career satisfaction (sample size varied across variables due to missing information). *χ* ^2^ tests

Variables	Total	Children (yes)	Children (no)	*P* value
Respondents (*n* (%))	102 (100)	60 (48.0)	42 (33.6)	
Motivation				
Better work-life balance				
Yes	32 (31.4)	26 (25.5)	6 (5.9)	0.002**
No	70 (68.6)	34 (33.3)	36 (35.3)	
Favorable consideration in the workplace				
Yes	49 (49.0)	42 (42.0)	7 (7.0)	< 0.001**
No	51 (51.0)	16 (16.0)	35 (35.0)	
Working experience as a full-time clinician				
Yes	84 (82.4)	53 (52.0)	31 (30.4)	0.06
No	18 (17.6)	7 (6.9)	11 (10.8)	
Presently work regularly as a part-time clinician				
Yes	50 (49.5)	35 (34.7)	15 (14.9)	0.03*
No	51 (50.5)	25 (24.8)	26 (25.7)	
Afterthoughts relinquishing full-time clinical practice				
Yes (often and sometimes)	33 (34.4)	24 (25.0)	9 (9.4)	0.02*
No (almost never or never)	63 (65.6)	30 (31.3)	33 (34.4)	
Academic rank				
Professor	15 (15.3)	7 (7.1)	8 (8.2)	0.6
Associate Professor	27 (27.6)	18 (18.4)	9 (9.2)	
Senior Assistant Professor	20 (20.4)	11 (11.2)	9 (9.2)	
Junior Assistant Professor	36 (36.7)	22 (22.4)	14 (14.3)	
Salary million JPY (thousand GBP)				
≤ 8.0 (63.0)	69 (70.4)	40 (40.8)	29 (29.6)	> 0.9
> 8.0 (63.0)	29 (29.6)	17 (17.3)	12 (12.2)	

*P < 0.05, **P < 0.01

The results of the binary logistic regression model showed that after adjusting for the influence of clinical experience as a full-time physician, there was still an association between childbirth experience and afterthoughts relinquishing full-time practice (*P* = 0.03) (Table 3).

$$ \log \left(p/1-p\right)=0.165X1+1.056X2\hbox{--} 1.019 $$

**Table 3:** Odds ratios for afterthoughts relinquishing full-time clinical practice by clinical experience and having children

Characteristics	Odds ratios	95% CI	*P* value
Clinical experience	1.2	0.4–3.8	0.8
Childbirth experience	2.9	1.1–7.2	0.03*

*P < 0.05

Binary logistic regression model

## Discussion

Research interest was the most frequent motivation for starting a career as a Basic Sciences physician (32.3%), reflecting an essential enthusiasm for exploring the unknown, first-hand, and advancing the frontiers of medicine [23]. As compared to female physicians without children, for those with children (1) work-life balance was more likely a motivation for entering Basic Sciences, (2) work-life balance was why Basic Sciences was considered an advantageous career choice for female physicians, and (3) favorable considerations such as a flexible schedule were offered in the workplace more often. These results suggest somewhat greater control over work-life balance for female physicians in Basic Sciences, consistent with findings from YY’s prior study [21]. A well-balanced lifestyle creates positive energy and motivation for women to continue careers, as professional fulfillment and responsibility for family life demand physical, emotional, and social resources [24]. One American sociologist suggests business and political leaders expect women to work like men [25]. In addition, the culture of computer science and technology heavily emphasizes total commitment to work at the exclusion of a personal life. Therefore, the proportion of Japanese women engaged in these areas is very low [25]. However, as more female workers enter these technological areas, males may become more inclined to balance personal life and career, shifting workplace culture. To sustain Japan’s medical workforce into the future, work-life balance is essential for both females and males.

Respondents with children were more likely to have clinical experience as full-time physicians, compared to respondents without children. However, the motivation for relinquishing full-time clinical work to enter the Basic Sciences was not requested, so a causal effect for the shift could not be ascertained. However, prior studies mentioned that female clinicians with children tended to change their workplace setting from academic hospitals to general hospitals, or from full time to part time, to balance work and family responsibilities effectively [26, 27]. In addition to a shift in workplace setting, it can be inferred that many female physicians would consider shifting their specialty from clinical medicine to Basic Sciences due to the demands of continuing clinical work after childbirth.

Female physicians with children were also more likely to engage in regular clinical work part-time. Expected income might not be the reason, since regular clinical work did not significantly increase income (*P* = 0.942). The ability to maintain a clinical position is useful for recruiting medical students to the Basic Science, since many shared the aim to be clinicians from the onset [21, 23]. However, the present study shows that about 84% of respondents had clinical experience but shifted from clinical medicine to Basic Sciences. Even with respondents who chose Basic Sciences as their primary specialty, 47% were engaged in regular clinical work, implying that Basic Scientists also value a clinician role, thereby appealing to medical students and young physicians who wish to see patients.

Generally, and especially in clinical fields, the number of females in the higher ranks is very small, regardless of commitments to career, family, and personal responsibilities [28]. Nomura and Gohchi noted that in Japan, gender disparity (the “glass ceiling”) still exists in medicine and may discourage career motivation among some female physicians [29]. In this study, the total number of professors was small (18), with 15% of respondents holding a rank of professor, and about half (46%) having children. Nonetheless, there was evidence that motherhood did not significantly affect respondents’ academic advancement or achieving professor rank in Basic Sciences, perhaps due to inherent flexibility in the research environment. Also, since productivity in publication is an important career marker for advancement in academic settings [30, 31], female physicians who regularly publish high-quality research can expect promotions, regardless of parenthood or “glass ceiling” limitations. Kvaerner et al. reported that female leadership in a medical specialty is associated with substantial increases in employment of women in the respective field [32]. Therefore, if female professors mentor female medical students or residents as a strategy for recruitment, greater interest in Basic Sciences could result.

Previous studies show that concerns about low salaries for Basic Sciences physicians create serious obstacles for medical students considering Basic Sciences as a career [21, 23]. In the present study, salaries in Japanese Yen (JPY) and British Pound (GBP) currency exchange rates as of October 2012 reveal that about 58% of respondents’ annual salary was between 8.0 million JPY (63.0K GBP) and 12.0 million JPY (94.5K GBP). In comparison, the average annual salary for clinicians working in general hospitals was about 15.0 million JPY (118.1K GBP), and for practitioners, about 25.0 million JPY (196.9K GBP) [33]. Considering income differences between Basic Sciences physicians and clinicians in general hospitals and clinics, a threshold professor-level salary of at least 15.0 million JPY (118.1K GBP) may be crucial to attracting students and young physicians to the Basic Sciences. Generally, however, clinicians’ salaries in academic hospitals are very low in Japan [34], with a study reporting the average annual income of a surgeon in one such location at 9.2 million JPY (72.4K GBP) for women and 11.3 million JPY (89.0K GBP) for men [34]. In academia, therefore, salaries of female Basic Sciences physicians may not be too much lower than female clinician salaries. Moreover, female clinicians with children tend to earn lower salaries than those without children [34]. However, in this study, even if age and academic rank are adjusted (*P* = 0.724), income levels did not significantly differ between female physicians with and without children, implying that parental duties may not affect career advancement for female physicians in the Basic Sciences.

Beyond favorability of Basic Sciences for career advancement, the study revealed that about 90% of respondents were satisfied with their career choice. Yet, regardless of clinical experience, female physicians in Basic Sciences fields who had children were more likely to express afterthoughts about relinquishing their full-time practice than non-parents. Perhaps Basic Sciences was a first choice for those without children, but for parents, a shift to Basic Sciences was likely an adaptation to family needs. Even within the academic ranks of assistant professor or higher, and with favorable considerations to facilitate parental duties, parents showed at least some remorse for their career shift. Japanese culture may have a much greater effect than the physician’s competence, productivity, or work environment. Already considered a common emotion among Japanese working mothers, the results of this study extends the sentiment to Japanese female doctors, regardless of specialty, with the same sense of inadequacy overshadowing past accomplishments.

Given women’s role of mother in Japanese culture [35], working mothers must convince others that they are “good mothers” despite their outside employment [36], with personal growth or career development often seen as a sign of selfishness during child-rearing [36]. Career obligations may lead to stigmatization of the individual [35], thereby precipitating change or abandonment of career.

Since Japan has provided universal healthcare insurance for over 50 years [37], the government manages healthcare [38], implementing personnel policies that are best served by strategies to tailor diverging career paths in the medical field before entry into medical school. At this point, the government has already devised two courses to alleviate physician shortages in certain areas: (a) a research physician course and (b) recruitment of clinicians who work in rural areas with applicants for medical schools [39]. Acknowledgment of the value, purpose, and support needs of female physicians who are parents is critical to future staffing of the healthcare system. Personnel crises can be better managed if pre-medical students are able to first consider which career path best suits their future: (1) full-time clinical, (2) full-time Basic Sciences, or (3) part-time clinical with part-time Basic Sciences. This pre-knowledge may improve satisfaction and retention.

This study has several limitations. First, subjects were identified as female and physicians by their feminine names and medical degrees, and their academic ranks were based on the university’s website. Therefore, if a first name could be applied to either gender, there was a possibility that questionnaires were sent to a few male physicians. However, the questionnaire clearly mentioned the survey’s specific focus on women, so male doctors would not be expected to answer questions, reducing the number of respondents. Second, some physicians in Basic Sciences fields do not consider themselves Basic Sciences physicians. For example, physicians in pathology or social medicines tend to think that they are not Basic Sciences physicians, because their specialties are more closely aligned to clinical fields than other Basic Sciences subjects. In addition, even if physicians are affiliated with Basic Sciences departments, sometimes their primary job is seeing patients or their position is temporary. Third, the response rate of this study was not particularly high (54.0%). Several factors contributed to the mediocre response rate. First, if a number of male physicians and non-physician female researchers mistakenly received questionnaires, or if the subject did not pertain to them, they likely did not respond. Second, websites of medical schools are not always updated, so questionnaires could have been sent to subjects no longer working in medical schools. Third, as the response rate of our previous study indicated [19], doctors tend to be extremely busy, with less time to respond to questionnaires. However, response was significantly higher than YY’s previous study on female clinicians, presenting with a 38.2% response rate [19]. Hence, response rate did not appear to weaken the quality of the study. Fourth, this study was conducted in Japan, and application of these results to other countries is limited in terms of different culture and educational system. Fifth, all subjects in the present study were ranked as assistant professor or higher. Therefore, respondents were career survivors who could manage work and private life in Basic Sciences fields. Responses from female physicians who left academia for any reason could not be gathered. Finally, this study is a cross-sectional study. To examine the causality, a longitudinal study is needed.

## Conclusions

The overwhelming majority of female physicians cited their interest in research as the reason for entering into a career of Basic Sciences. Given that motivations to choose medicine as a career path include an outstanding aptitude in medical science, a substantial work ethic, and a fervent desire to improve community health [23, 40], these outcomes are an expected result. Female doctors join the many young professionals with families to seek work-life balance. Even with many expressing afterthoughts relinquishing full-time clinical practice, job flexibility is an attractive benefit to choosing a Basic Sciences career. Due to looming shortages among Basic Sciences physicians, boosting medical students’ interest in research through lectures, practical trainings, and mentorship, as well as providing insight into advantages which include work-life balance, may help the field to attract more medical students, regardless of a student’s gender.

In this study, reasons were not examined for the afterthoughts that female physicians with children expressed in shifting to Basic Sciences when relinquishing a full-time clinical practice. Possible causes, such as duty over choice, provide a need for future study. This manuscript examined female physicians’ dilemmas in balancing roles as a good mother and a good doctor from Japanese cultural aspects. Japanese women’s issues are seen differently from Westerners’ standards, and these problems should be examined in their indigenous cultural context [36]. As with other nations, gender, race, ethnicity, and social status may affect a medical doctor’s specialty choices [41–43]. Yet, regardless of social and cultural barriers, medical educators and policymakers should respect the interests and curiosity of potential medical school candidates, providing support for a student’s ultimate career path [43] with guidance and motivation, which are so crucial for long-term satisfaction and career retention [43].

## Additional file

Additional file 1: A questionnaire survey about female physicians in the Basic Sciences. (DOCX 13 kb)
