# Supplementary material for: Professional fulfillment and parenting work-life balance in female physicians in Basic Sciences and medical research: a nationwide cross-sectional survey of all 80 medical schools in Japan
Source: Hum Resour Health. 2017 Sep 15;15:65. doi: 10.1186/s12960-017-0241-0 (PMC5602846; doi:10.1186/s12960-017-0241-0)
Supplement: Additional file 1: — A questionnaire survey about female physicians in the Basic Sciences. (DOCX 13 kb) [file 12960_2017_241_MOESM1_ESM.docx]

**Additional file**

A Questionnaire survey about Female Physicians in the Basic Sciences

Please choose the most suitable number or describe your opinion in the blank space.

Also, please describe your answer in the blank parentheses.

1．Indicate your current age.

　　1）20’s　　 2）30-34　　3）35-39　　4）40-44　　5）45-49

　　6）50-54　　7）55-59　　8）60-65　　9）Over 66

2．How many years between graduation from medical school and the time you entered Basic Sciences department, regardless of position?

　（　　　　）years

3．Choose the reasons why you entered Basic Sciences fields (Multiple choices allowed).

1）Interest in research

2）Interest in disorders

3）Continued in Basic Sciences after completing Ph.D. Degree.

4）Wanted to promote my own career.

5）Wanted to leave clinical work.

6）No particular reason, by chance.

7）Better work-life balance

8）Others（　　　　　　　　　　　　　　　　　　　　　　　　　　）

4．What is your specialty? ( )

5．What is your position?

1）Full-time assistant professor 2）Full-time instructor

3）Full-time associate professor　 4）Full-time professor

5）Other（　　　　　）

6．Have you ever had full-time clinical experience, including residency?

1)　Yes　　　2）No

7. Do you engage in clinical work regularly?

1)　Yes　　　2）No

8．Do you feel that you should have become a clinician?

1）Yes, often　　　2）Yes, sometimes　　　3）Almost never 4) Not at all

9. Do you have children?

1）Yes　　　　2）No

10-1．Do you think that Basic Sciences is a good fit for female physicians?

1）Yes　　2）Generally Yes　　3）Not sure　4）Generally No　　5）No

10-2． If you answered “Yes” or “Generally yes” in the previous question, please choose the reasons. (Multiple choices allowed)

1）Having time to take care of children

2）Not physically demanding

3）Better work-life balance

4) Basic Sciences fits women’s personality.

5）Other（　　　　　　　　　　　　　）

11. Have you received any special considerations because you are a woman after you entered Basic Sciences departments?

1)　Yes　2) No　3) Not sure

➥Please describe your experiences.

(　　　　　　　　　　　　　　　　　　　　　　　　　　　　)

12. Do you make an effort to inspire students to develop an interest in research when giving lectures or practical trainings?

1) Yes　　2）Generally Yes　　3) Generally No 4) No

5) Not engaged in lectures or practical trainings

13. Are you satisfied with your present job?

1) Yes　　2) Generally Yes　　3) Not sure 　4)　Generally No　 5) No

14. Insufficient salaries for Basic Scientists are the biggest concern, which discourages medical students from entering Basic Sciences fields.

If you do not mind answering this question, would you mind telling us your total salary from last year (including salary from part-time jobs)? Include the tax (Currency Unit: million JPY).

1) < 4.0 2) 4.0-6.0 3) 6.0-8.0 4) 8.0-10.0

5) 10.0 -12.0 6) 12.0-14.0 7) 14.0-16.0 8) 16.0-18.0

9) 18.0-20.0 10) > 20.0
